# Applicability and costs of nanofiltration in combination with photocatalysis for the treatment of dye house effluents

**DOI:** 10.3762/bjnano.5.55

**Published:** 2014-04-15

**Authors:** Wolfgang M Samhaber, Minh Tan Nguyen

**Affiliations:** 1 Institute of Process Engineering, Johannes Kepler University Linz, Welser Strasse 42, A-4060 Leonding, Austria; 2 Institute for R&D of Natural Products, Hanoi University of Science and Technology, 1 Dai Co Viet Road, Hanoi, Vietnam

**Keywords:** dye industry effluent, environmental, membrane, nanofiltration, photocatalysis, UV

## Abstract

Nanofiltration (NF) is a capable method for the separation of dyes, which can support and even improve the applicability of photocatalysis in effluent-treatment processes. The membrane process usually will need a special pre-treatment to avoid precipitation and fouling on the membrane surface. Conceptually NF can be applied in the pre-treatment prior to the catalytic reactor or in connection with the reactor to separate the liquid phase from the reaction system and to recycle finely suspended catalysts and/or organic compounds. When concerning such reaction systems on a bigger scale, cost figures will prove the usefulness of those concepts. Different applications of photocatalysis on the lab-scale have been published in recent years. Membrane technology is used almost in all those processes and an overview will be given of those recently published systems that have been reported to be potentially useful for a further scale-up. NF membranes are mostly used for the more sophisticated separation step of these processes and the additional costs of the NF treatment, without any associated equipments, will be described and illustrated. The total specific costs of industrial NF treatment processes in usefully adjusted and designed plants range from 1 to 6 US$/m^3^ treated effluent. Combination concepts will have a good precondition for further development and upscaling, if the NF costs discussed here in detail will be, together with the costs of photocatalysis, economically acceptable.

## Introduction

Textile processing comprises different operations such as pre-treatment, dyeing, washing of garments, printing and finishing and produces a large amount of polluted effluent. For processing one ton of textile, 230 to 270 m^3^ of wastewater has to be treated prior to the release into the environment [1]. Conventional biological treatment plants are not effective in the removal of colour dye effluents, because of the aromatic structure of the large dye molecules, which provides chemical stability and, thus, also a high resistance to biological degradation. Dyes are made to be stable to light, oxidizing agents, and aerobic digestion to fulfil the quality demands of textile products. Fundamental principles and applications of photocatalytic degradation of dyes in homogeneous or heterogeneous systems can be found in the literature. For example there is an extensive overview given from Mills and Le Hunte [2], a review by Chong et al. [3] about recent developments in photocatalytic water treatment technology, and a short description of fundamentals is given by Rauf and Salman Ashraf 2009 [4].

## Results

### Conventional concepts of effluent treatment and NF

In the conventional treatment of effluent of the textile industries separation methods like coagulation, flocculation, flotation or sedimentation are used. Process variants concerning the separation of dyes are numerous, but all of them require a final disposal, possibly with a prior on-site storage. The drawbacks of all chemical methods are an additional usage of chemicals, an increased sludge production and often the need to remove additional colour and chemical oxygen demand (COD). Unconventional membrane separation plants with NF filters, in combination with certain pre-treatment steps, are applied in some textile works, but those nanofiltration applications do not yet reflect the state of the art, and are not yet adopted as standard techniques in the dye industry [5]. However, the major drawback of applying membrane processes in wastewater treatment is membrane fouling. Therefore a proper pre-treatment of the feed of a nanofiltration separation is the most important measurement to obtain a successful technical application. Many reports are found in the literature, which deal with possible measures for the prevention of fouling in membrane filtration. One recent review focuses the coagulation in connection with nanofiltration has been published by Zahrim et al. [6]. Flocculation and the separation of flocculated materials can reduce COD and colour to a great extent and, in addition, decrease the fouling rate in a membrane process.

**Figure 1:**
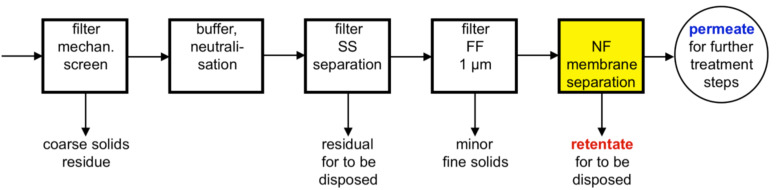
General pre-treatment steps prior to membrane separation.

In Figure 1, a schematic overview of possible pre-treatment steps is displayed and it can be recognised that pre-treatment plays an important role but will also be costly when membranes are applied in the wastewater treatment. In the nanofiltration permeate, which constitutes the bigger part of the treated effluent, COD and colour are usually reduced to a great extent. If necessary, an additional NF step can be installed further down-stream as a post-treatment process.

### Membrane filtration combined with photocatalysis

Photocatalysis is an advanced method for the degradation of dyes from textile effluent due to its ability to oxidize and to destruct dyes simultaneously while the conventional treatment methods either concentrate or transfer dyes to a solid phase [7–11]. Fundamentally, organic compounds are decomposed by means of reactive species such as hydroxyl radicals (OH^•^), which are generated by UV irradiation of photocatalysts in the reaction system. Commonly applied photocatalysts include TiO_2_, ZnO, Fe_2_O_3_, CdS, GaP and ZnS. Among these, titanium dioxide (TiO_2_) has attracted great interest in research and development because of its mechanical properties, chemical and thermal stability and resistance to chemical breakdown, which promote its application in photocatalytic water treatment [7,9,12–13]. Photocatalysts can be used in the form of suspended fine particles or immobilized on various supports. Obviously, photoreactors with a suspended catalysts (or slurry type) are considered to offer greater contacting surfaces between the photocatalysts and the pollutant molecules than reactors working with immobilized photocatalysts. Immobilized catalysts have a defined specific surface area, which is connected with the supporting surface. Photoreactors with suspended catalysts, however, require a separation of the catalyst from the treated effluent.

The combination of photocatalysis and membrane filtration is based on the fact that photocatalysts exhibit an oxidation potential and the membrane separation, especially with nanofiltration membranes, provides the selective separation of pollutants to be retained and removed. Different concepts have been described in literature. The use of particulate catalysts, require a recirculation of the catalyst, and it is in addition necessary to uncouple the hydraulic residence time from the residence time of the organic compounds in the catalytic reactor system, which can be achieved by selective membrane separation [3,10,12,14–16]. Photocatalytic processes have been predominantly selected to be combined with pressure-driven membrane processes such as microfiltration (MF) [17], ultrafiltration (UF) [10,17–18], nanofiltration (NF) [5,10,19–21] and reverse osmosis (RO) [19]. Recently, the combination with membrane distillation (MD) [10,18] has also been proposed for the treatment of dye industry effluents.

Molinari et al. [21] studied the degradation of two commercial azo-dyes, namely Congo red (C_32_H_22_N_6_Na_2_O_6_S_2_) and patent blue (C_27_H_31_N_2_NaO_6_S_2_), by using TiO_2_ Degussa P25 as the photocatalyst in a lab-scale combined system with NF membranes NTR 7410 (Nitto Denko, Tokio) and have observed that it was possible to successfully treat concentrated solutions (500 mg/L) of both dyes by means of a continuous process with a suspended photocatalyst. Damodar et al. [17] have studied the coupling of a MF membrane separation with a photocatalytic laboratory slurry reactor for an advanced treatment of dye effluent and achieved high removal rates (82–100% colour removal, 45–93% TOC removal, and 50–85% COD removal) at optimal initial concentration of reactive black 5 (RB5) in a flat polytetrafluoroethylene (PTFE) MF membrane module submerged into the slurry photocatalytic reactor. Moreover, the submerge membrane concepts enabled long-term test runs. Grzechulska-Damszel et al. [10] investigated the removal of azo dyes (acid red 18, direct green 99 and acid yellow 36) from water in different combined systems: (a) photocatalysis with immobilized catalyst bed/NF and (b) photocatalysis in suspension/UF/MD. Berberidou [19] achieved a complete decolourization of a synthetic dye effluent containing reactive black 5 with a combined system of photocatalysis and RO/NF, as well as a more than 95% reduction of the initial organic content and salinity. Mozia [8] conducted experiments with two combined systems: photocatalysis–ultrafiltration and photocatalysis–membrane distillation for the degradation of acid red 18 in an aqueous solution. Both membrane processes could achieve a separation of TiO_2_ from the solution. The MD process separated the model dye completely while the UF process only removed 77% of the dye after 5 h of the irradiation. Photocatalysis and membrane processes in combination can also be accomplished with various photocatalytic membrane reactors (PMRs), many of which have been described in the literature [3,16–17,21]. PMRs can generally be divided into two main groups: fixed-bed photoreactors and slurry batch photoreactors. Molinari et al. [22] compared different PMRs in terms of the position of UV irradiation. Irradiation can take place in the flat sheet membrane cell or in a separated recirculation loop. Different configurations were applied for both fixed-bed photoreactors and slurry batch photoreactors. The authors indicated that an advantage of the system with the suspended photocatalyst over the fixed one is to avoid the risk of a possible membrane oxidation by OH^•^ radical attack, because the photocatalytic reaction is effectively separated from the membrane filtration step.

### NF concepts with photocatalysis

**Figure 2:**
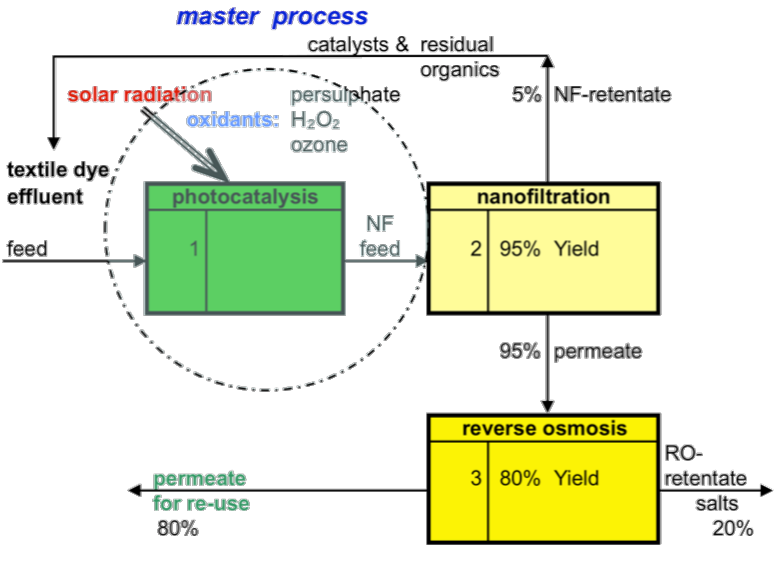
Photocatalysis as the master process responsible for the reduction of organic and fouling matter with a nanofiltration and RO-step for further removal of residual contaminants.

To combine a nanofiltration process with photocatalysis there are two basic concepts to consider. NF can be set on the up-stream side or on the down-stream side of the photocatalytic reactor. In Figure 2 the photocatalysis process is shown as the responsible step to achieve or fulfil the main process requirements. The NF step down-stream from the photocatalysis reactor operates for the recycling of catalysts and residual organic compounds and will achieve additional improvements in the quality of the treated effluent streams. The permeate of the NF step can optionally be fed to a reverse osmosis (RO) step in order to separate the salts from the relatively well purified NF permeate stream to produce water for re-use.

Zheng et al. [5] have investigated the colour removal and COD reduction in biologically treated dye effluent. With submerged NF hollow fibres, it was possible to remove 99.3% of colour and 91.5% of COD while maintaining a steady permeat flux of 5.15 L/m^2^·h with an applied trans-membrane pressure of 0.8 bar. Colour compounds of biologically pre-treated effluent could be separated by NF separation, which would enable the combination with a photocatalytic reactor, in which the reject stream could be treated in parallel. From this example, it can be concluded that these concepts require, on the one hand, a feed that is not too highly loaded and has a sufficient transparency for the photocatalytic reaction. On the other hand, the drain from the photocatalysis will possibly contain less fouling matter with the advantage that the NF can be operated with a significant reduction of membrane fouling. The challenge of this concept can further be seen in its highly efficient oxidation. The following NF acts almost solely as a polishing step and possibly for the recovery or recycling of the catalysts. A clear effluent stream with less complex constituents together with a high optical transmission is favourable for such a concept. Finally, it must be kept in mind that the further quality improvements through NF and RO will have to justify the costs of the additional membrane steps down-stream.

**Figure 3:**
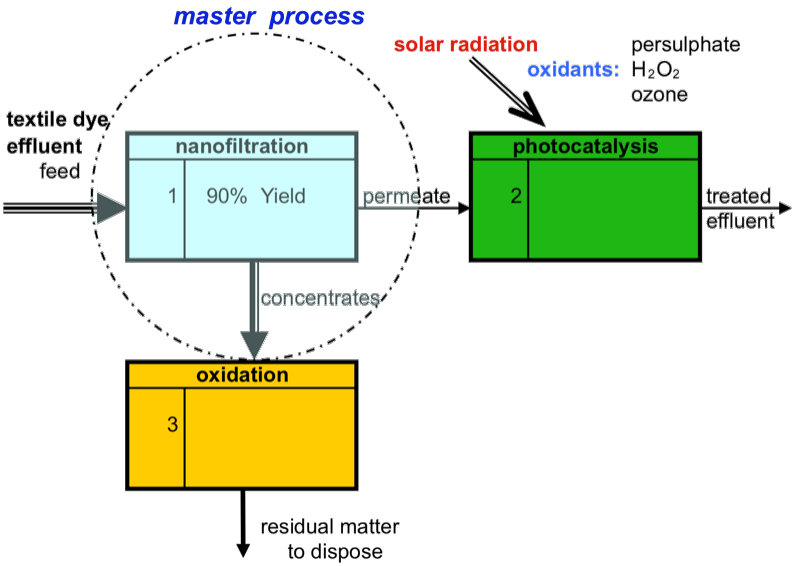
Nanofiltration as the master process responsible for the major reduction of colour and organic compounds, while photocatalysis acts as a polishing step.

Another way of combining NF and photocatalysis is shown in Figure 3. In this schematic block diagram, the NF step is located on the up-stream side of the photocatalysis and therefore the NF is primarily responsible for the separation. In other words, NF will be the master process and other processes down-stream, such as the photocatalysis, are connected and/or adjusted with or to the NF. Here, photocatalysis acts more or less as a polishing step to reduce residual colour compounds, which are contained in the NF permeate. This concept is comparable with a general NF treatment concept at the source, and therefore, the feed has to be pre-treated in order to avoid membrane fouling as mentioned previously. Fouling of membranes is often a weakness of the membrane process and the development of a proper pre-treatment recipe is therefore a challenging task as described earlier.

The retained dyes and organic matter are separated and represent the concentrate or retentate stream, which have to be treated further and finally disposed according to local regulations. The permeate stream, which is already reduced in colour and in dissolved organic compounds, is post-treated in the photocatalysis, which can again be classified as a polishing step.

**Figure 4:**
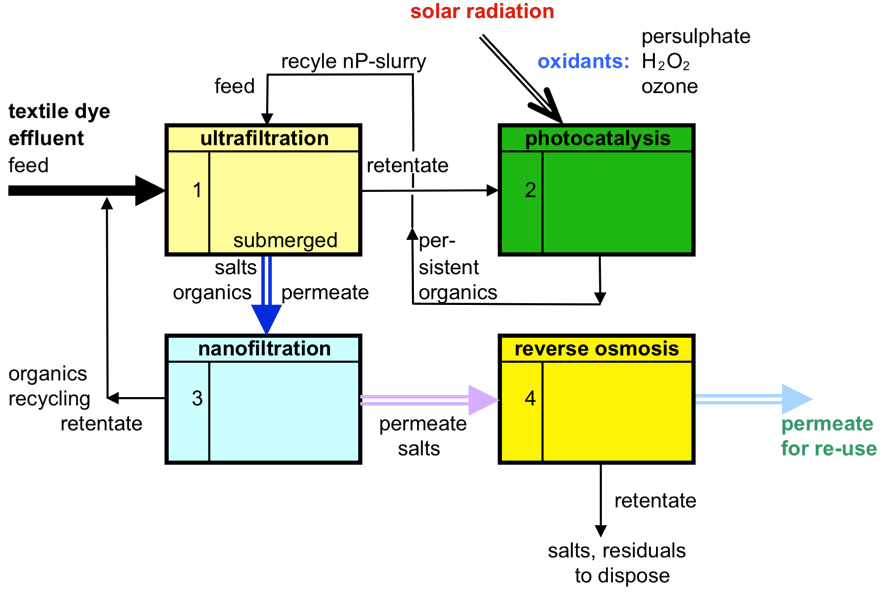
Photocatalysis combined with a submerged UF separation and a down-stream NF and RO treatment.

A more sophisticated concept is shown in Figure 4. A submerged UF membrane is used to keep the nano-sized catalyst particles within the UV-radiated reaction chamber. A comparable system is described by Patsios et al. [23] for the continuous degradation of humic acids. In a heterogeneous catalysis with TiO_2_ the successful removal of 5 to 10 mg/L humic acid from a synthetic effluent, without any reject stream, was demonstrated.

If effluent of the dye industry is processed, there will be organic colour and inorganic salts in the feed stream. In the backflow of the photocatalytic system, compounds of not yet reacted or oxidized organic matter are collected. The UF retentate is returned back to the photocatalytic reaction system. The UF permeate will be used as the feed to the down-stream NF separation step. Retained compounds from the nanofiltration will be recycled to the UF feed. The NF step should be arranged in such a way, that the residence time of persistent organic compounds could be increased within the system. The NF permeate again can be treated optionally to separate salts and minor residuals down-stream within an RO step. The main feed of the dye effluent will need respective pre-treatment, after which the effluent stream has to be transparent and clarified and to contain only a minor content of dyes and dissolved organic compounds. The photocatalytic process can be carried out almost without a rejected fraction and no further disposal of residual matters.

### Estimation of NF costs

The synergies of applying nanofiltration in combination with photocatalysis must justify the additional costs. As we have seen, NF can be a pre-treatment step to increase the effectiveness of the photocatalysis or can act as a post-treatment after the photocatalysis, for a further reduction of colour and COD. In both process concepts, the NF will contribute significantly to the dye separation, but it will also be a major contribution in the treatment costs. For a rough and a quick estimation of these costs, a simple approach will be described and demonstrated in an example. The described procedure is based on the authors experiences in the realization of NF-plants for the production of dyes and chemicals and for the pre-treatment of wastewater in the 1980s and 1990s, within the dye division of Sandoz, as well as for intermediates and solvents isolation for DSM and Evonik in recent years. According to those experiences, the costs of the NF treatment can be attributed to the membrane replacement costs, which directly depend on the required membrane area and therefore, on the size of the plant that is used for the treatment process. When membrane plants are applied, it is a challenge to keep membrane costs low, because of the frequent need for membrane replacement that is associated with these applications. Membrane costs in industrial applications are in the range of 10 to 20% of the total equipment costs. In wastewater treatment the maximum affordable membrane replacement costs (MRC) are, as a rule, less than 10% of the equipment costs.

The annual operating costs, as given in Table 1, are between 204 and 408 US$, which is roughly seven times the assumed membrane replacement costs (MRC) of 30 to 60 US$ per m^2^ of the spirally wound membrane elements used in the focused treatment plant. The estimated figures of fixed and variable costs are empirical cost data collected from NF membrane plants with membrane areas from 100 to 500 m^2^ with a cost accuracy of ±30%, depending on the quality, technical performance and efficiency of the separation. Based on the figures of Table 1, we can roughly estimate the operating cost of an NF application, which has to achieve a given through-put of permeate. The permeate flux indicated in Table 1 as the volumetric specific permeate capacity, which empirically will be in the range between 10 to 30 L/m^2^·h for an NF-application.

**Table 1:** Compilation of costs in multiples of membrane replacement costs.

specific membrane replacement costs (sMRC) for spiral membrane elements		30–60 US$/m^2^
volumetric permeate capacity		5–30 L/m^2^·h
assumed membrane life time (MLT)		1 a

**fixed costs**	amortization	2.55 × MRC
	maintenance (20% of amortization)	0.50 × MRC
	
	total fixed costs	3.05 × MRC

**variable costs**	membrane costs	1.00 × MRC
	energy costs	0.50 × MRC
	cleaning (CIP) costs	0.25 × MRC
	labour costs	2.00 × MRC
	
	total variable costs	3.75 × MRC

**total operating costs**		**6.80 × MRC**

A short example illustrates this cost estimation procedure. The first steps of this approach are shown in Table 2. We assume an application with a treatment capacity of 20 m^3^/d and 200 d per year of operation. For cleaning (CIP) 4 h per day is set and the net operating time per day results in 20 h. With the assumed 200 operating days per year, we get the specific permeate capacity between 200 and 600 L/m^2^·d or, when calculated for one year, of 40 to 120 m^3^/(m^2^·a). Depreciation and maintenance costs incur independently of the operatinal status of the plant. That is, no matter whether or not there is a demand to treat effluents, the periodical CIP is included in the maintenance cost, which is required, even if the membrane plant is not in operation.

**Table 2:** Cost estimation example based on Table 1 for a treatment capacity of 20 m^3^ dye effluent per day at an assumed operation time of 200 days per year.

dye effluent	20 m^3^/d
specific permeate capacity	10 L/(m^2^·h)
operation	20 h/d
specific permeate capacity per day	200 L/(m^2^·d)
resulting size of NF plant	100 m^2^ (membrane area size, MAS)
sMRC	30–60 US$/m^2^ (mean value: 45 US$/m^2^)
membrane lifetime	1 a
total sMRC (100 m^2^ MAS)	3,000–6,000 US$/a
cost estimation of Table 1 (365 d/a operation)	6.80 × MRC
fixed costs (Table 1)	3.05 × MRC
variable costs (Table 1) for 200 d/a	2.05 × MRC (= 200/365 × 3.75 MRC)

**total operating costs**	**15,300–30,600 US$/a (= 5.10 × MRC)**

**Figure 5:**
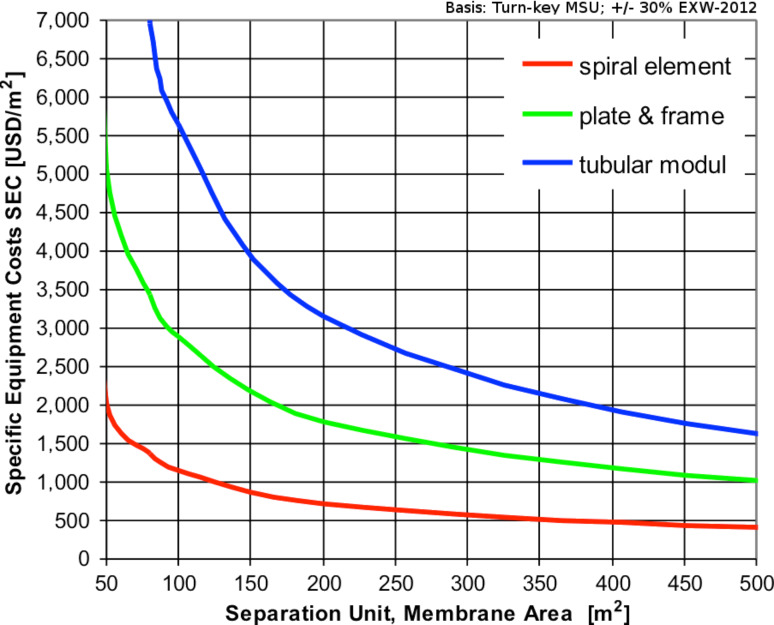
Specific equipment cost (SEC) per m^2^ of membrane area.

As for the amortization period, we have generously taken a 10-year period, which might not be generally applicable depending on local situations or financial regulations. Figure 5 is a compilation of empirical data of realized membrane plants with different module configurations such as tubular, plate and frame, or spiral wound. The given specific equipment costs are the turn-key costs of frame-mounted separation plants, including the CIP system, without the costs for local installation of buffer tanks and all out-side the battery limits of the separation plant, which are considered to be ex-works prices. From Figure 5 we can now take the estimated purchase price for the equipment cost, based on the necessary plant size, which is defined by the membrane area needed for the filtration process and based on the design output mentioned previously.

The required plant size in our example is defined with a 100 m^2^ membrane area. As we are using spiral wound configuration, the specific equipment costs can be taken from Figure 5 to be about 1,150 US$/m^2^, which results in an equipment purchase price of 115,000 US$ (±30% depending quality of materials, instrumentation, control devices and process automation standards, etc.). As calculated in Table 2, the total operating cost will be 15,300 US$ (for 3,000 US$ membrane replacement cost) or 30,600 US$ (for 6,000 US$ MRC), respectively. With those figures we can calculate the treatment cost of one cubic metre of dye effluent, which results, depending again on the respective membrane replacement cost, in the range between 3.83 and 7.66 US$/m^3^.

### Influences on NF treatment costs

**Figure 6:**
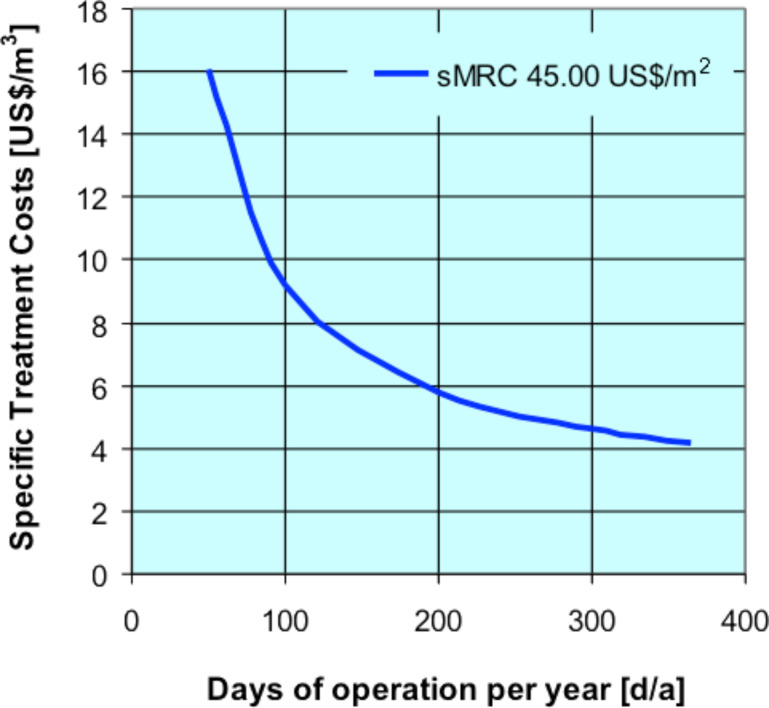
Total specific treatment costs of dye industry effluent versus the operating time in d/a with a mean MRC value of 45 US$/m^2^.

The daily required effluent treatment capacity and time for membrane cleaning together with the main specific permeate flux, should be taken for the preliminary fixing of the NF plant size, as illustrated in the previous example, whereby influences in flux performances are not taken in consideration. With the amortization costs the invested capital of a plant, which can be estimated on the basis of the plant size, will be paid off over a certain period time. The number of years for paying off the capital expenditures is one cost sensitive factor and another is given with the yearly operating hours. This influence affects to a great extent the total specific treatment costs. The total mean treatment cost (MRC = 45 US$/m^2^) can be calculated as 5.74 US$/m^3^, for our example of a NF treatment plant with a capacity of 20 m^3^ dye effluent per day and with an assumed membrane flux of 10 L/m^2^·h and 200 operating days per year, which means that 4,000 m^3^ would be treated during one year. Those treatment costs would be decreased to 4.20 US$ /m^3^ if the plant could be operated the whole year with a total treatment capacity of 7,300 m^3^. The number of treatment days per year, which represents a significant influence in the treatment costs too, is shown in Figure 6.

**Figure 7:**
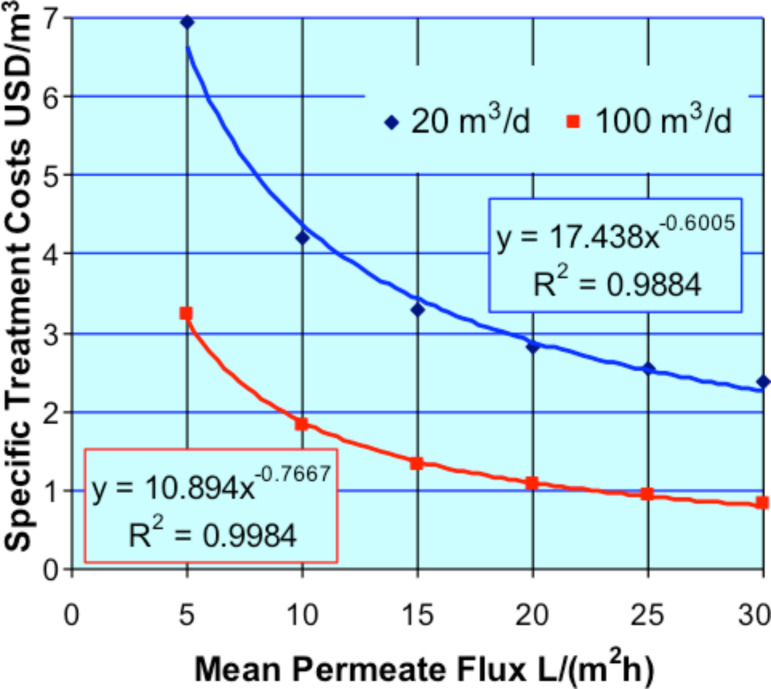
Specific total operating costs, treatment capacity 20 m^3^/d and 100 m^3^/d, 365 days per year treatment operation.

Even a well-performing plant has to be preferably operated round the year for a profitable application. A treatment plan over the year is needed to reduce down-time periods and overcapacities of treatment plants. Overcapacities and long down-times can kill the economic benefits of a projected treatment plant. Alongside these effects, CIP time and/or membrane cleaning times, together with the achievable permeate fluxes, will have an additional influence on the demandable sizes of the membrane plant areas and represent therefore another significant factor in the separation plant and costs. In Figure 7, the total specific treatment costs in US$ per treated cubic metre effluent is shown versus the mean plant permeate flux which can be achieved in the considered treatment process. In Figure 5, the dependence of the specific investment or equipment costs versus the membrane areas or plant sizes is displayed. Taking into account this and an assumed amortization period of 10 years, the total specific treatment costs are calculated for an assumed 365 days per year operation, and for plant capacities of 20 m^3^/d and 100 m^3^/d. In Figure 7, the resulting treatment costs in US$ per cubic metre of dye effluent are outlined versus the achievable mean permeate flux of the NF process.

## Discussion

Focusing on industrial applications it is a rule that costs are associated almost directly with the numbers of treatment steps involved. To combine different treatment steps it is important to learn about the strengths and weaknesses of the single steps to be able to evaluate the opportunity of a certain combination. In our case photocatalysis is used to degrade compounds in the effluent. The nanofiltration is used to isolate compounds selectively from the effluent. The combination of the two has to ensure the required removal of dyes from the effluent, without generating a reject stream which would again require a further treatment step or has to be finally disposed. The assessment of the applicability of nanofiltration in combination with photocatalysis is clearly given in the treatment application, if the constituents of effluents can be rejected or isolated by the nanofiltration as well as properly degraded by the photocatalysis.

The costs of the nanofiltration separation, which have to be taken in account, dependent on different empirical factors, which can be obtained from respective experiences or have to be specifically collected through lab and/or pilot tests, which should be done preferably in combination with the projected photocatalytic reactor. Regarding the cost calculation of nanofiltration, assumptions were made, which are based as far as possible on practical experiences. The expected specific treatment costs lie in the range between 1 and 6 US$ per m^3^ of treated effluent depending on plant size, quality of effluent to be treated and required treatment limits.

## Conclusion

The combination of NF with photocatalysis is capable of increasing the efficiency of the dye degradation process. NF possesses the ability to reject organic colour compounds in the pre-treatment, as well in the post-treatment. Photocatalysis needs a more transparent system and therefore lower concentration of dyes to be effectively applied. Despite their application potential NF membrane processes are not a common technology in dye works, yet. Promising laboratory results of NF separation are not easily transferred to industrial applications, and it has to be kept in mind that membrane processes are seldom stand-alone solutions and additional investment costs for industrial plants that include the necessary pre-treatment equipment can be high.

Considering the treatment of dye effluents, there is a possibility to set the treatment at the source to avoid the disadvantage of a dilution of dyes being treated. Previously many dyehouses discharged their effluents to the main sewer and, as a consequence, the treatment of the collected dye effluents had to be carried out in large-volume tanks. Secondly various residual dyes from different sources have to be separated or oxidized more or less at the end of the pipe after a biological treatment step to fulfil regulations or to achieve an almost colourless effluent. The treatment of diluted systems after the biological degradation, also described as effluent polishing, can be carried out conventionally through natural UV-radiation in large surface ponds or, with reduced area demands, in photocatalytic systems, in which the so-called advanced oxidation processes are conducted. The photocatalytic systems exhibit higher efficiencies and shorter residence times and nanofiltration can contribute by almost completely rejecting organic compounds, which are not readily degraded within the given hydraulic residence time in the photocatalysis. However, a nanofiltration down-stream of the photocatalytic reaction will be a major cost factor. The exclusive costs of NF will range from 1 to 6 US$ per m^3^ of treated effluent. But as a result, NF will ascertain high qualities of the treated effluents and can be synergistically combined with a photocatalytic degradation facility.
